# Gluten serological testing in various dog breeds with paroxysmal dyskinesia

**DOI:** 10.3389/fvets.2023.1119441

**Published:** 2023-03-03

**Authors:** Casey B. Rogers, Nina Meyerhoff, Holger A. Volk

**Affiliations:** Department of Small Animal Medicine and Surgery, University of Veterinary Medicine Hannover, Hannover, Germany

**Keywords:** gluten sensitivity, gliadin, transglutaminase, movement disorder, neurology

## Abstract

**Background:**

Paroxysmal gluten-sensitive dyskinesia is a subtype of movement disorder classified as canine paroxysmal dyskinesia (cPD), which until now has only been described in Border Terriers (BT).

**Objectives:**

Our aim was to report cPD with positive gluten serology in dog breeds other than BT.

**Animals:**

Thirty-one client-owned dogs with suspected cPD were examined in this study.

**Methods:**

The hospital records of the dogs where the serum was tested for modified gliadin peptide immunoglobulin G (gliadin IgG) and tissue transglutaminase-2 immunoglobulin A (transglutaminase-2 IgA) were studied. A total of 31 dogs were presented to the clinic with cPD. A work-up consistent with Tier 1 or Tier 2 confidence levels for canine epilepsy was undertaken in all dogs. The dogs' diets and episode descriptions or videos in 16/31 cases were additionally studied. A follow-up was held to inquire about the dogs' wellbeing and response to the diet changes.

**Results:**

Fourteen of the 31 dogs tested positive for gluten sensitivity with either gliadin IgG or transglutaminase-2 IgA or both ratios elevated. In seven dogs, serology was classified as questionable with gliadin IgG or transglutaminase ratios mildly elevated. Ten dogs tested negative. According to the owners' reports, five of the dogs that tested positive had no more episodes after changing to a strictly gluten-free diet, with one of the dogs relapsing twice after being fed treats containing gluten. Three dogs had a reduction in episode frequency of >50%, and two dogs had shorter and less intense episodes.

**Conclusion:**

A considerable subset of dog breeds presented for presumed cPD showed laboratory signs of gluten sensitivity and responded to a gluten-free diet.

## Introduction

Paroxysmal gluten-sensitive dyskinesia (PGSD) has been diagnosed in Border Terriers (BT) (1–3). It falls under the subtype of movement disorders classified as canine paroxysmal dyskinesia (cPD). Canine paroxysmal dyskinesias consist of episodes during which affected dogs often show episodic changes of posture or involuntary movements, with or without increased muscle tone, typically without autonomic signs such as hypersalivation or urination and without loss of consciousness (4). In BT with PGSD, associated gastrointestinal signs and borborygmus have been reported in about 50% of dogs (5). Episodes last from minutes to hours and can occur in clusters or be interspersed by months (5). Clinical and neurological examination as well as magnetic resonance imaging (MRI) and cerebrospinal fluid (CSF) analysis are unremarkable in primary cPD and PGSD (2). A clinical diagnosis relies on the exclusion of structural causes for paroxysmal episodes (5) and other paroxysms. However, differentiation from focal epileptic seizures can be challenging. Epileptic seizures are defined as manifestation(s) of excessive synchronous, usually self-limiting epileptic activity of neurons in the brain (6). This results in a transient occurrence of signs which may be characterized by short episodes with convulsions or focal motor, autonomic, or behavioral features due to abnormal excessive and/or synchronous epileptic neuronal activity in the brain (6).

The role of gluten in cPDs was first described in the study of Lowrie et al. in 2015, in which serum antibodies were detected in BT with cPD. The serum antibodies used to test for gluten sensitivity in dogs are modified gliadin peptide immunoglobulin G (gliadin IgG) and tissue-transglutaminase-2 immunoglobulin A (transglutaminase-2 IgA) (7). These are reliable markers to diagnose coeliac disease in people (8) and have since been successfully implemented for diagnosing PGSDs in BT in combination with responses to diet changes (1). Coeliac disease (CD) is an immune-mediated enteropathy, which occurs in genetically susceptible individuals upon ingestion of gluten (9, 10). Neurological disorders such as gluten ataxia (GA) are part of an extraintestinal spectrum of related conditions (11). In people with GA, only 40% have associated enteropathy, and therefore, fall under the term of noncoeliac gluten sensitivity (12). In these patients, gliadin antibodies are considered the best diagnostic markers for gluten sensitivity (13, 14).

To the authors' knowledge, laboratory findings in PGSDs have only been described in BT and PGSD has clinically been suspected in Maltese dogs with cPD improving under a gluten-free diet (13). The occurrence of PGSD in other breeds is unknown. Our hypothesis for the current study was that PGSDs occur in breeds other than BT. We aimed to report the percentage of dogs with cPD with an increased gliadin IgG and transglutaminase-2 IgA. The results of this study could improve the decision for therapy options in clinical practice when dogs present with cPD.

## Materials and methods

The retrospective monocentric study was conducted at the Small Animal Hospital of the University of Veterinary Medicine Hannover. Digital hospital records^1^ were searched for the time period from October 2019 (the date of the first available commercial gluten serology testing for dogs in Germany) to July 2022 using the search terms listed in Supplementary Table 1.

**Figure 1:**
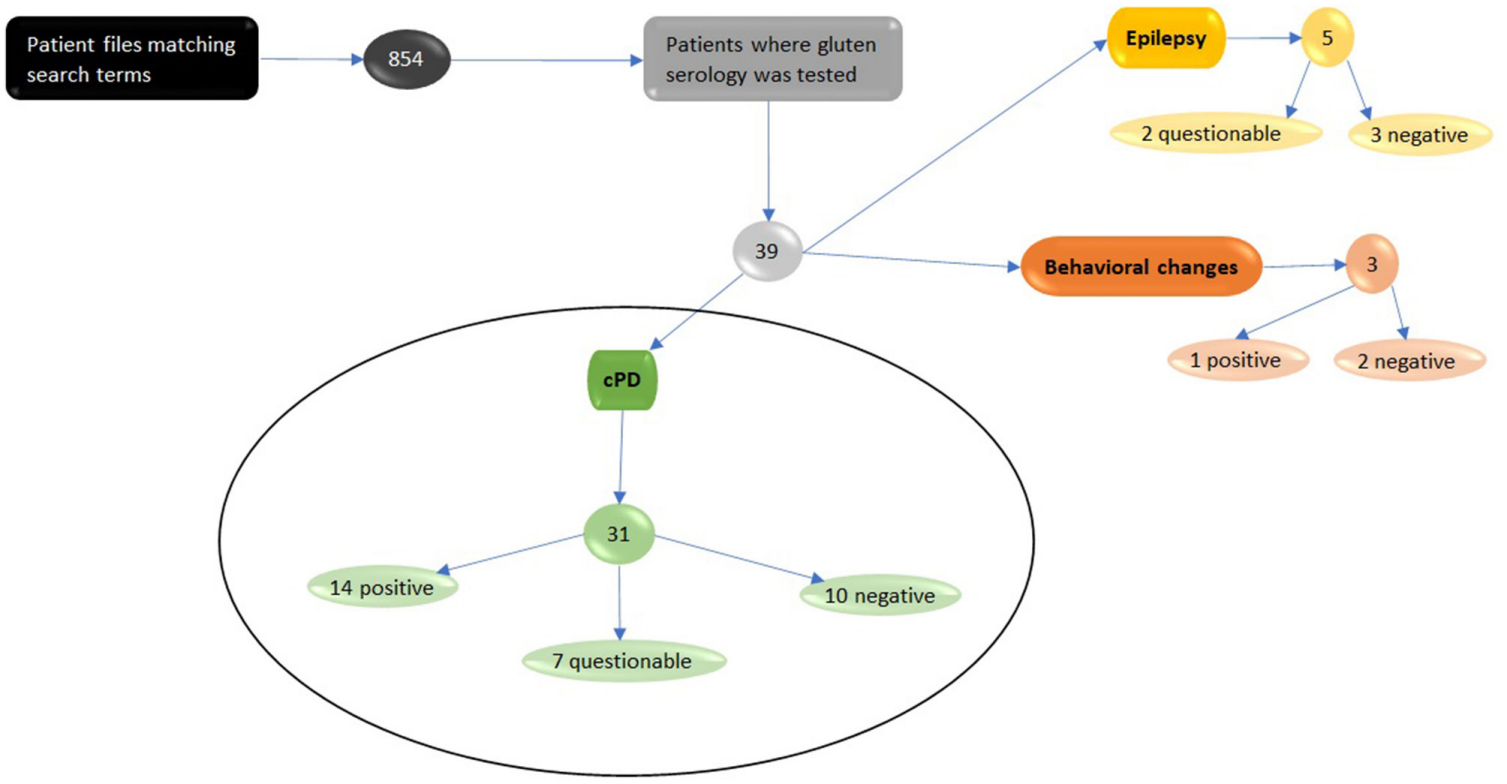
Eight hundred and fifty-four digital patient files were found using search terms depicted in Supplementary Table 1. The files were studied and thirty-nine dogs were found that had been tested for serological evidence of gluten sensitivity markers. Thirty-one of those dogs were initially diagnosed with canine paroxysmal dyskinesia and therefore included in this study. Fourteen tested positive for gluten-sensitivity, seven questionable with slightly elevated markers and ten tested negative. We excluded five dogs with suspected Epilepsy, of which two had slightly elevated marker levels and three tested negative. The three dogs that were tested due to behavioral changes where also excluded. Of the three dogs one tested positive and two negative.

Consequently, 854 patient files containing the search terms were reviewed. Of those, all canine cases with testing of gluten serology were included in the study. A total of 39 cases where a gluten-sensitivity test was initiated were identified and reviewed in detail. A total of 31 of those dogs were presented with suspected cPD (Figure 1), and the remaining cases were subsequently diagnosed with behavioral disorders or idiopathic generalized epilepsy.

Each dog underwent a TIER 1 or TIER 2 work up recommended for canine epilepsy (15). Additionally, a serum sample of every dog was sent to a commercial laboratory^2^ to be tested for serological evidence of gliadin IgG and transglutaminase-2 IgA using a standardized and commercially available ELISA test. The laboratory categorized the results as positive (ratio > 0.8) and questionable (ratio 0.6–0.8) if either transglutaminase-2 IgA or gliadin IgG results were within the range of a ratio between 0.6 and 0.8. A negative result was classified with both gliadin IgG and transglutaminase-2 IgA results at a ratio <0.6.

Information regarding diet was extracted from the hospital records including detailed nutrition questionnaires. Episode characteristics were reported by the owners and in 20/37 cases, videos taken by the owners were reviewed. Follow-up for the 31 patients was conducted *via* phone call or e-mail communication with the dog's caretakers. Only descriptive statistics are presented here using a commercial software package.^3^

## Results

### Animals

A total of 31 dogs were included in this study, 13 were female (5/13 neutered) and 18 male (10/18 neutered). The median age at disease onset was 4 years (range 1–9 years) and 5 years and 6 months of age at presentation (range 2–12 years). Various breeds and mixed-breed dogs were included in this study, which can be found in Table 1.

**Table 1:** Sex, breed, weight, and age at presentation and episode onset of the 31 dogs, categorized by gluten-sensitivity test results into positive, questionable, negative, and total.

**Sex**	**Positive serology**	**Questionable serology**	**Negative serology**	**Total**
Female	4	3	1	8
Female, neutered	1	0	4	5
Male	5	2	1	8
Male, neutered	4	2	4	10
**Breed**				
Mixed breed	5	2	4	11
Standard poodle	1	0	0	1
Cairn Terrier	1	0	0	1
Rhodesian Ridgeback	1	1	0	2
Russian Terrier	1	0	0	1
Perro de Agua espanol	1	0	0	1
Shetland Sheepdog	1	0	0	1
Pomeranian	1	0	1	2
Border Terrier	1	0	0	1
Norwich Terrier	0	1	0	1
Akita Inu	0	1	0	1
Chihuahua	0	1	0	1
Poodle	0	1	1	2
Beagle	0	0	1	1
Podenco	0	0	1	1
Pug	0	0	1	1
Bichon Frise	0	0	1	1
Australian Shepherd	1	0	0	1
Total	14	7	10	31
**Weight (kg)**				
<10 kg	6	2	6	14
10–20 kg	4	3	3	10
20–30 kg	2	0	1	3
>30 kg	2	2	0	4
Median (range)	16.06 (2.0–35.5)	17.5 (3.0–45.6)	10.05 (2.8–23.6)	14.45 (2.0–45.6)
**Age at presentation (years)**				
Median (range)	5 (3–11)	6.4 (2–12)	5.1 (1.5–8)	5.5 (2–12)
**Age at onset (years)**				
Median (range)	4.2 (1.5–9)	3.9 (1–8)	3.7 (1–8)	4 (1–9)

### Diet

Various veterinary prescription diets, commercial diets available without a prescription containing gluten, as well as gluten-free diets and homemade raw diets were fed prior to testing for gliadin IgG and transglutaminase-2 IgA (Table 2). Commercial gluten-free diets use gluten-free ingredients but, in contrast to prescription gluten-free diets, do not guarantee a gluten-free production.

**Table 2:** Reported diet per dog (*n* = 31) in which gluten serology was tested before consultation in the small animal hospital.

**Diet pre-consultation**	**Positive dogs**	**Questionable dogs**	**Negative dogs**	**Total**
Commercial containing gluten	4	1	4	9
Commercial gluten free	6	3	1	10
Commercial unknown gluten status	2	1	1	4
Veterinary prescription containing gluten	1	0	0	1
Veterinary prescription unknown gluten status	0	1	2	3
Homemade raw diet	1	1	2	4
Information not available	0	1	0	1
Treats containing gluten	1	0	0	1

Dogs were categorized depending on the gluten sensitivity test result into dogs testing negative, questionable, or positive for gluten sensitivity markers. In total, 33 answers for 31 patients were listed as two dogs received a combination of diets.

### Episodes

The dogs presented to the clinic were seen due to having episodes characteristic of movement disorders. Episode frequency ranged from daily clusters to dogs having one episode every few months and durations ranged from few seconds up to 90 min. Further information and detailed frequency and episode length, as well as the onset and triggers for episodes of each dog, can be found in the dataset for this study.

The most common signs shown during episodes involved gait or limb muscle tone abnormalities and changes in posture. Forty-eight percent (*n* = 15/31) of the dogs showed loss of limb control with dystonia making it the most represented clinical sign. Other signs involving the whole body or limbs include body tremors in 39% (*n* = 12/31), increased muscle tone and muscle contractions of various muscles or groups of muscles in 35% (*n* = 11/31), and an arched back in 16% (*n* = 5/31) of dogs. Athetosis and swaying of the trunk were each seen in 6% (*n* = 2/31) of dogs and one dog (3%) showed an increased extensor tone. Ataxia was seen in 35% (*n* = 11/31) of dogs and one dog appeared disorientated during the episodes (3%). Some dogs showed clinical signs involving the head during episodes, which ranged from head tremors in 11% (*n* = 3/31) and head bobbing in 6% (*n* = 2/31) of dogs to the wide swaying of the head, the head turning to both sides, and myokymia, which were each seen in one of the 31 dogs (3%).

Episode onset varied and appeared when calm in 22% of dogs (*n* = 7/31), after stress or excitement in 13% of dogs (*n* = 4/31), or when sleeping in 3% of dogs (*n* = 1/31). Thirty-nine percent of dogs (*n* = 12/31) had episode onsets randomly, with owners not being able to make out specific times or situations in which the dogs showed episodes. In 22% of dogs (*n* = 7/31), there was no information available regarding the onset.

Detailed episode descriptions and clinical signs or behavior prior to and post episodes for each dog individually can be found in the dataset for this study. The phenotype of episodes related to the detection of gluten serology (gluten-sensitive positive, questionable, and negative dogs) is depicted in Supplementary Tables 2–4.

### Investigations

Abnormal results in the minimum blood database were only found in one of the 31 dogs, which showed slightly elevated bile acids (39.7 μmol, reference range 0–20 μmol), and 30/31 dogs' results were unremarkable, including the bile acid results in the remaining 30 dogs. Further diagnostics included MRI + CSF (*n* = 9/31), urine analysis (*n* = 17/31), abdominal ultrasound (*n* = 4/31), T4 and TSH serum levels (*n* = 7/31), electromyography (*n* = 2/31), blood pressure examination (*n* = 1/31), and otoscopic examination (*n* = 1/31). Abnormal results in further diagnostics include one dog (*n* = 1/9), which showed abnormalities in the MRI with a Chiari-like malformation typical for the breed.

### Concurrent treatment

Prior to consultation or post-consultation, 13/31 dogs received no medications. Medication or supplements were received by 18/31 dogs including imepitoin (*n* = 7/18), levetiracetam (*n* = 5/18), levothyroxine-natrium (*n* = 2/18), phenobarbital (*n* = 1/18), topiramate (*n* = 1/18), taurine (*n* = 1/18), propentophylin (*n* = 1/18), nicotinamide (*n* = 1/18), L-carnitin (*n* = 1/18), mesalazin (*n* = 1/18), B-vitamins (containing thiamine, riboflavin, and cobalamin) (*n* = 1/18), acetazolamide (*n* = 1/18), and fluoxetin (*n* = 1/18). Detailed information on combinations, dosage, and prescription prior to consultation or post-consultation can be found in the dataset for this study.

### Concurrent disease

No concurrent disease was reported in 25/31 dogs. In six of the 31 dogs, concurrent disorders including hypothyroidism (*n* = 2/6), inflammatory bowel disease (*n* = 1/6), corneal dystrophy (*n* = 1/6), symmetrical lupoid onychodystrophy (*n* = 1/6), spondylosis in the thoracic and lumbar vertebral column (*n* = 1/6), suspected Euthyroid Sick Syndrome (*n* = 1/6), food sensitivity (*n* = 1/6), and chronic bladder stones (*n* = 1/6) were reported.

### Gluten sensitivity

The serum of 31 dogs was tested for gliadin IgG and transglutaminase-2 IgA. Fourteen dogs tested positive with either gliadin IgG (range 0.123–2.536, median 0.743) or transglutaminase-2 IgA (range 0.400–1.625, median 0.842) or with both ratios elevated. In seven dogs, serology was classified as questionable with gliadin IgG (range 0.330–0.470, median 0.441) or transglutaminase-2 IgA (range 0.567–0.792, median 0.706) ratios mildly elevated. Ten dogs tested negative for gliadin IgG (range 0.163–0.540, median 0.362) and transglutaminase-2 IgA (range 0.207–0.500, median 0.335); therefore, yielding normal results.

### Response to diet change

The median time between the consultation and follow-up for all dogs was 7 months (range 1–27 months). According to the owners' reports, five (*n* = 5/14) of the dogs with abnormal gluten serology showed complete cessation of episodes after changing to a strictly gluten-free diet, with one of the dogs relapsing twice by showing cPD episodes shortly after being fed gluten-containing treats. Of these five dogs, four were put on a strict gluten-free diet, using commercial gluten-free food and owners cut out gluten-containing treats. The remaining dog was introduced to a veterinary prescription gluten-free diet. Three (*n* = 3/14) dogs had a reduction in episode frequency of >50% after the introduction of a strict, commercial gluten-free diet (*n* = 2/3) or a prescription gluten-free diet (*n* = 1/3). Two (*n* = 2/14) dogs had no reduction in episode frequency but show shorter and less intense episodes after the introduction of a commercial gluten-free diet. Two (*n* = 2/14) owners did not change their dog's diets, one (*n* = 1/14) dog had a diet change too recent to the follow-up, one (*n* = 1/14) dog had no reported response to changing from a commercial gluten-free diet to a strict veterinary prescription gluten-free diet. In the dogs with questionable gluten serology without concurrent pharmacological treatment, two dogs (*n* = 2/7) had no further episodes after changing to a strict, commercial gluten-free diet. Both dogs relapsed once after ingesting gluten and showed episodes of cPD. From the dogs that tested negative, one owner reported that after changing to a gluten-free diet episodes resolved completely, despite a negative test result and no concurrent treatment.

## Discussion

The purpose of this study was to investigate the percentage of dogs with cPD with an increased serum gliadin IgG and transglutaminase-2 IgA, and therefore, the possible occurrence of PGSDs in dog breeds other than BT. Positive gluten serology was found in 14 of 31 tested dogs (45%) with cPD and questionable antibody ratio in nine (29%) dogs which, therefore, could be considered as patients possibly suffering from PGSD. However, due to the retrospective nature of the study causing inhomogeneity of reported clinical signs, a final diagnosis could not be made in several cases. To fully rule out epileptic seizures, identification of electroencephalographic abnormalities characteristic of seizure disorders would be necessary (15, 16). Nevertheless, this study highlighted the importance of the gut–brain axis (GBA) and dietary effects on the clinical outcome of cPD.

The GBA is a highly complex interactive network between the gut and the brain which works *via* neuroendocrine, immune, and inflammatory pathways (17, 18). In idiopathic epilepsy (IE) and neurodegenerative diseases, studies have shown the relationship between the gut and brain and the importance of the microbiome and gut health on disease pathogenesis, susceptibility, and treatment (17–21). In 36% of serologically positive cases (*n* = 5/14) a solely dietary treatment resulted in complete remission of episodes according to the owner's statements. It should be investigated further to what extent diet, and therefore, the GBA plays a role in diseases like PGSDs in other breeds than BT. Any form of treatment that improves the patients' and owners' quality of life could be considered a success. Further investigations, therefore, should be made into the effects diet changes have on the compliance and quality of life in owners and patients suffering from PGSDs, possibly reducing the use of pharmaceuticals in patients that are treatable with diet changes. This should take into account that a possible placebo effect, as seen in dogs suffering from IE, cannot be ruled out (22), especially in the one serological negative case, in which a diet change led to the cessation of cPD. Additionally, spontaneous remission has been reported in certain breeds with cPD (23, 24).

The pathogenesis and involvement of the immune system should be further researched for canine PGSD; a positive result in gliadin IgG and transglutaminase-2 IgA could be caused by other immunological reactions and not necessarily be solely gluten induced. The clinical relevance of slightly elevated gliadin IgG and tissue-transglutaminase-2 IgA in dogs is uncertain and needs further research. In people, positive Transglutaminase-2 IgA results have been reported in patients suffering from autoimmune diseases such as type 1 diabetes, autoimmune liver disease, Hashimoto's thyroiditis, psoriatic or rheumatoid arthritis, and heart failure, who do not have celiac disease (25–27). Furthermore, currently unknown antibodies or metabolites may play a role in PGSD, which could explain the positive outcomes to diet changes in dogs that tested negative for gliadin IgG and transglutaminase-2 IgA. The role of gliadin IgG and transglutaminase-2 IgA in dogs with IE is also unclear, opening the field to further studies.

Although not further discussed in this study, the authors want to mention one dog that presented with behavioral changes due to abnormal gluten serology. This may also be caused by gluten sensitivity, as seen before in a case report by Suñol et al. in 2020 (28). In people, psychological manifestations and neurocognitive impairments have been linked to CD (29, 30). However, due to the lack of diet change and unavailable follow-up, this can only be speculated in this case.

There are several limitations to the current study related to the study design. The data we used were obtained retrospectively, which lead to less controlled data collection and thus a great variation in external influences. The clinical signs and episode descriptions were inhomogeneous and difficult to define due to variations in accounts in the digital records. Diets and especially diet changes were not controlled, and therefore, the results were subjective, which was taken into account upon interpretation. In addition, due to the uncontrolled diet change and a small number of dogs put on a veterinary prescription gluten-free diet, a comparison between the effectiveness of commercial vs. prescription gluten-free diets could not be made. For future studies, re-examining gliadin IgG and transglutaminase-2 IgA after diet changes and possibly doing diet challenges by reintroducing gluten in patients could also improve the interpretation of positive results to said dietary adjustments. A further limitation was the possibility that there may have been dogs with focal epileptic seizures among the dogs diagnosed with cPD, as no EEG testing was performed to fully rule in or out canine epilepsy, as recommended in the IVETF consensus guideline for a Tier 3 confidence level in canine idiopathic epilepsy (15).

Despite not all the dogs having a positive test result or a positive response to a gluten-free diet, the results of this study show the potential that a long-term reduction in the use of medication as treatment is possible for some patients. In conclusion, a considerable subset of various dog breeds presented for presumed cPD showed laboratory signs of gluten sensitivity as well as response to treatment consisting of diet changed. Clinicians presented with cPD cases should consider PGSD as a possible underlying cause in various dog breeds.

## Data availability statement

The raw data supporting the conclusions of this article will be made available by the authors, without undue reservation.

## Author contributions

CR, NM, and HV conceived the study, participated in its design and coordination, and wrote the final version of the manuscript. CR and NM collected the data. CR performed the statistical analysis, which was verified by HV and both authors interpreted the data obtained. All authors have read and approved the final manuscript.

## Footnotes

^1^Easyvet.

^2^Laboklin GMBH & CO.KG, Labor für Klinische Diagnostik, Dr. Elisabeth Müller (Firmenleitung), Adresse: Steubenstraße 4, 97688 Bad Kissingen, Deutschland.

^3^Microsoft Excel Spreadsheet.
