# Supplementary material for: Gluten serological testing in various dog breeds with paroxysmal dyskinesia
Source: Front Vet Sci. 2023 Mar 3;10:1119441. doi: 10.3389/fvets.2023.1119441 (PMC10020495; doi:10.3389/fvets.2023.1119441)
Supplement: Supplementary file 1 [file Data_Sheet_1.docx]

# Supplementary Material

| **Table 1 search terms used to find digital patient files matching cPD cases** | |
| --- | --- |
| **Search terms** | Gluten-Antibodies  Dyskinesia  Paroxysmal  Gliadin  Transglutaminase  Gluten-Sensitivity  Movement disorder  Seizure/ seizures  CNS-disease  Hereditary ataxia of Terriers  Ataxia  Tremor /shaking  Focal epilepsy |

Table 2 clinical signs during, before and after cPD episodes of the dogs tested positive for gluten sensitivity serum markers in amount and percentage of dogs showing the signs.

| Gluten serology positive dogs | | |
| --- | --- | --- |
| **Clinical signs during episode** | Number of dogs (14 total) | Percentage |
| Loss of limb control with dystonia | 8 | 57% |
| Increased muscle tone and muscle contractions | 6 | 43% |
| Body tremor | 6 | 43% |
| Ataxia | 5 | 36% |
| Head tremor | 2 | 14% |
| Arched back | 2 | 14% |
| Head bobbing | 1 | 7% |
| Swaying of the head | 1 | 7% |
| Swaying of the trunk | 1 | 7% |
| Athetosis | 1 | 7% |
| Questionably conscious | 1 | 7% |
| ***Autonomic signs*** |  |  |
| Vomiting | 2 | 14% |
| Salivation | 2 | 14% |
| Urination | 2 | 14% |
| **Behavior prior to episode** | | |
| Normal | 8 | 57% |
| Clinginess | 3 | 21% |
| Information not available | 3 | 21% |
| **Behavior / clinical signs post episode** | | |
| Normal | 8 | 57% |
| Clinginess | 1 | 7% |
| Exhaustion | 3 | 21% |
| Diarrhea | 1 | 7% |
| Vomiting | 1 | 7% |
| Information not available | 3 | 21% |

Table 3 clinical signs during, before and after cPD episodes of the dogs with moderately elevated (=questionable) gluten sensitivity serum markers in amount and percentage of dogs showing the signs.

| Gluten serology questionable dogs | | |
| --- | --- | --- |
| **Clinical signs during episode** | Number of dogs (7 total) | Percentage |
| Increased muscle tone and muscle contractions | 3 | 43% |
| Loss of limb control with dystonia | 2 | 29% |
| Arched back | 2 | 29% |
| Ataxia | 2 | 29% |
| Tremor body | 2 | 29% |
| Aggression | 2 | 29% |
| Swaying of the trunk | 1 | 14% |
| Head bobbing | 1 | 14% |
| Increased extensor tone | 1 | 14% |
| Myokymia | 1 | 14% |
| ***Autonomic signs*** |  |  |
| Salivation | 2 | 29% |
| **Behavior/ clinical signs prior to episode** | | |
| Normal | 2 | 29% |
| Agitation | 1 | 14% |
| Aggression | 2 | 29% |
| Clinginess | 1 | 14% |
| Information not available | 1 | 14% |
| **Behavior/ clinical signs post episode** | | |
| Normal | 2 | 29% |
| Disorientation | 1 | 14% |
| Clinginess | 2 | 29% |
| Anxiety | 1 | 14% |
| Nausea | 1 | 14% |
| Exhaustion | 1 | 14% |
| Information not available | 1 | 14% |

Table 4 clinical signs during, before and after cPD episodes of the dogs tested negative for gluten sensitivity serum markers in amount and percentage of dogs showing the signs.

| Gluten serology negative dogs | | |
| --- | --- | --- |
| **Clinical signs during episode** | Number of dogs (10 total) | Percentage |
| Loss of limb control with dystonia | 4 | 40% |
| Ataxia | 4 | 40% |
| Tremor body | 4 | 40% |
| Increased muscle tone and muscle contractions | 2 | 20% |
| Tremor head | 1 | 10% |
| Right sided drift | 1 | 10% |
| Head turn to both sides | 1 | 10% |
| Arched back | 1 | 10% |
| Dystonia in the hind limbs | 1 | 10% |
| Disorientation | 1 | 10% |
| Athetosis | 1 | 10% |
| ***Autonomic signs*** |  |  |
| urination | 1 | 10% |
| defecation | 1 | 10% |
| vomiting | 1 | 10% |
| **Behavior/ clinical signs prior to episode** | | |
| Normal | 7 | 70% |
| Vomiting | 1 | 10% |
| Salivation | 1 | 10% |
| Restlessness | 1 | 10% |
| Information not available | 1 | 7% |
| **Behavior/ clinical signs post episode** | | |
| Normal | 4 | 40% |
| Ataxia | 1 | 10% |
| Anxiety | 1 | 10% |
| Vomiting | 1 | 10% |
| Exhaustion | 3 | 30% |
| Disorientation | 1 | 7% |
| Tremor | 1 | 7% |
| Information not available | 1 | 7% |
